# Ribosomal DNA loci derived from *Brachypodium stacei* are switched off for major parts of the life cycle of *Brachypodium hybridum*

**DOI:** 10.1093/jxb/ery425

**Published:** 2018-11-27

**Authors:** Natalia Borowska-Zuchowska, Ewa Robaszkiewicz, Elzbieta Wolny, Alexander Betekhtin, Robert Hasterok

**Affiliations:** Department of Plant Anatomy and Cytology, Faculty of Biology and Environmental Protection, University of Silesia in Katowice, Katowice, Poland

**Keywords:** 35S rDNA, 35S rRNA, allopolyploidy, *Brachypodium hybridum*, embryo tissues, FISH, meiosis, nucleolar dominance, nucleolar organizing region

## Abstract

Nucleolar dominance is an epigenetic phenomenon that occurs in some plant and animal allopolyploids and hybrids, whereby only one ancestral set of 35S rRNA genes retains the ability to form the nucleolus while the rDNA loci derived from the other progenitor are transcriptionally silenced. There is substantial evidence that nucleolar dominance is regulated developmentally. This study focuses upon the establishment and/or maintenance of nucleolar dominance during different stages of development in the model grass allotetraploid *Brachypodium hybridum*. Fluorescence *in situ* hybridization with a 25S rDNA probe to cells in three-dimensional cytogenetic preparations showed that nucleolar dominance is present not only in root meristematic and differentiated cells of this species, but also in male meiocytes at prophase I, tetrads of microspores, and different embryonic tissues. The inactive state of *Brachypodium stacei*-originated rDNA loci was confirmed by silver staining. Only *B. distachyon*-derived 35S rDNA loci formed nucleoli in the aforementioned tissues, whereas *B. stacei*-like loci remained highly condensed and thus transcriptionally suppressed. The establishment of nucleolar dominance during earlier stages of *B. hybridum* embryo development cannot be ruled out. However, we propose that gradual pseudogenization of *B. stacei*-like loci in the evolution of the allotetraploid seems to be more likely.

## Teaser

Analysis of 35S rDNA activity in meiocytes, microspores, and different stages of embryo development in *Brachypodium hybridum* reveals that *Brachypodium stacei*-like loci are switched off.

## Introduction

The 35S/45S rRNA genes, which encode the precursor of the three largest ribosomal RNAs (18S, 5.8S, and 25–28S rRNA), are among the most-studied genes of eukaryotic genomes. 35S rDNA units are present in most plant genomes, while the 45S rDNA is characteristic of animals (Mirzaghaderi *et al.*, 2017). Despite the fact that there are hundreds to thousands of tandemly repeated 35S rDNA units per genome, spanning millions of base pairs along the chromosomes, not all genes are actively transcribed (Lewis and Pikaard, 2001). The number of active genes depends on the demand of a cell for ribosome and protein synthesis, which changes dynamically during plant development (Mohannath *et al.*, 2016). Superfluous 35S rRNA genes undergo silencing via reversible epigenetic modifications, such as DNA methylation and dimethylation of lysine K9 in histone H3 (H3K9me2) (Bockor *et al*., 2014; Costa-Nunes *et al.*, 2014). Moreover, the number of DNA-dependent RNA polymerase I (PolI) enzyme molecules that transcribe the 35S rRNA genes is regulated (French *et al.*, 2003; McStay and Grummt, 2008). Only loci that contain actively transcribed rRNA genes, known as nucleolar organizing regions (NORs), are able to form a nucleolus. Interestingly, transcriptionally active and suppressed rDNA units belonging to the same locus occupy different subnuclear compartments. The actively transcribed genes are present within the nucleolus, whereas the silenced genes are located adjacent to the nucleolus and bear epigenetic modifications characteristic of heterochromatin (Pontvianne *et al.*, 2013; Borowska-Zuchowska and Hasterok, 2017). However, the epigenetic and transcriptional states of particular rDNA units are fully interchangeable according to the needs of the cell (Pontvianne *et al.*, 2013).

In some animal and plant interspecific hybrids and allopolyploids, a whole set of 35S rRNA genes inherited from one progenitor can be selectively suppressed (Pikaard, 2000; Ge *et al.*, 2013). This phenomenon, originally described as ‘differential amphiplasty’ (Navashin, 1934), is now known as nucleolar dominance (ND). The inactive state of one ancestral set of rDNA loci in a hybrid/allopolyploid organism, in which ND is established, is maintained via the same chromatin-mediated repression mechanisms that are responsible for the rRNA gene dosage-control in non-hybrid species (Lewis and Pikaard, 2001; Dobesova *et al*., 2015). For instance, it was shown in wheat–rye hybrids and *Brassica* allotetraploids that the under-dominant rRNA genes can be reactivated by inhibitors of both DNA methylation [5-azacytidine (5-AzaC) and 5-azadeoxycytidine (5-AzadC)] and histone deacetylation [trichostatin A (TSA)] (Vieira *et al.*, 1990; Amado *et al.*, 1997; Chen and Pikaard, 1997*a*). More detailed studies of ND in the allotetraploid *Arabidopsis suecica* revealed the involvement of an RNA-dependent DNA methylation (RdDM) pathway and concerted DNA methylation, histone methylation and histone deacetylation changes in ND enforcement (Lawrence *et al.*, 2004; Earley *et al.*, 2006; Preuss *et al.*, 2008; Costa-Nunes *et al.*, 2010). However, the exact molecular mechanisms that determine which ancestral set of rRNA genes is to be transcriptionally silenced remain obscure. ND in plants is independent of any maternal effect or parental rDNA copy number, and may be developmentally regulated (Chen and Pikaard, 1997*b*). The hypotheses that differential expression of rRNA genes corresponds to either physical characteristics of rRNA gene intergenic spacers (IGSs) or differences in binding affinities of transcription factors do not apply to plants (Chen and Pikaard, 1997*b*; Frieman *et al.*, 1999). More recent studies on diploid *Arabidopsis thaliana* confirmed the role of a chromosomal position effect, which was previously proposed by Nicoloff *et al.* (1979), in the selective suppression of rDNA loci (Mohannath *et al.*, 2016).

Idziak and Hasterok (2008) documented ND for the first time in root meristematic cells of the allotetraploid *Brachypodium hybridum* (2n=30; genome composition BdBdBsBs; Lusinska *et al.*, 2018). Two diploid annual representatives of this genus, *Brachypodium distachyon* (2n=10; BdBd) and *Brachypodium stacei* (2n=20; BsBs), closely resemble the ancestral species of *B. hybridum*. Recently, Borowska-Zuchowska *et al.* (2016) showed that the *B. stacei*-like 35S rDNA loci are suppressed not only in root tip cells but also in differentiated cells of *B. hybridum* roots. They also reported that the rDNA loci inherited from the two ancestors had distinct and different epigenetic modifications: *B. distachyon*-like loci are enriched in euchromatic histone modifications (e.g. H4K5ac, H4K16ac, and H3K9ac) and the *B. stacei*-inherited rDNA loci have the heterochromatic marker H3K9me2 and a high level of DNA methylation. Interestingly, the demethylation of DNA by 5-AzaC treatment did not reactivate the *B. stacei*-like loci (Borowska-Zuchowska and Hasterok, 2017).

In the current study, we used complex cytogenetic approaches to determine whether the preferential suppression of *B. stacei*-inherited 35S rDNA loci in *B. hybridum* is a reversible, developmentally regulated process. We analysed the spatial distribution of 35S rDNA loci in prophase I meiocytes and microspores of *B. hybridum* using three-dimensional (3D) cytogenetic preparations. We show that the highly condensed *B. stacei*-like loci do not form a nucleolus at the beginning of meiotic division and in microspores. The inactive state of these loci was confirmed directly by silver staining of meiocytes at pachytene. Additionally, we show that *B. stacei*-like loci at pachytene have significantly higher levels of DNA methylation compared with those of *B. distachyon*. Furthermore, the inactive state of *B. stacei*-derived 35S rDNA loci is maintained in all embryonic tissues of *B. hybridum* at two distinct developmental stages.

## Materials and methods

### Plant material

Seeds of *B. hybridum* Catalán, Joch. Müll., Hasterok & Jenkins genotype ABR113 were obtained from the collections held by the Institute of Biological, Environmental and Rural Sciences, Aberystwyth University, UK. They were sown at high density in pots with soil mixed with vermiculite (3∶1 w/w), and grown at 22 °C and with a 16/8 h light/dark photoperiod in a greenhouse (ABR113 does not require vernalization and usually starts flowering approximately 4 weeks after planting). Immature inflorescences for the analysis of meiosis were collected and fixed in fresh 3:1 ethanol:glacial acetic acid at room temperature (RT). After 24 h, the fixative was refreshed and the material was stored at –20 °C until use. Additionally, *B. hybridum* embryos were collected at two developmental stages, BBCH01 and BBCH83 according to the Biologische Bundesantalt, Bundessortenamt and Chemische Industrie (BBCH) scale for *B. distachyon* (Hong *et al.*, 2011). Dry seeds with embryos at the BBCH00 stage were placed in a Petri dish on moist filter paper at RT in the dark for 4 h. Whole seeds with embryos (at the beginning of seed imbibition; BBCH01 stage) were then fixed in fresh 3:1 ethanol:glacial acetic acid at RT for 2 h. Embryos at BBCH83 were collected from green, immature spikes ~2 weeks after pollination and were fixed in 3:1 ethanol:glacial acetic acid, as described above.

### Squashed meiotic chromosome preparations

Meiotic chromosomes from anthers were prepared according to Jenkins and Hasterok (2007). Briefly, individual anthers were isolated, washed in 10 mM citric acid–sodium citrate buffer (pH 4.8) and digested enzymatically in a mixture comprising 10% (v/v) pectinase (Sigma), 0.65% (w/v) cellulase Onozuka R-10 (Serva), 0.5% (w/v) cellulase (Calbiochem), 0.15% (w/v) cytohelicase, and 0.15% (w/v) pectolyase in 10 mM citrate buffer at 37 °C for ~2 h. Three or four anthers of similar size were transferred to a slide in a drop of 45% acetic acid, covered with a coverslip, and squashed. After freezing on dry ice, the coverslips were gently removed and the slides were air-dried.

### Slide preparation from embryos

Embryos at BBCH01 and BBCH83 were isolated manually and dehydrated in an ethanol:2×saline–sodium citrate (SSC) buffer series (75%, 90%, 95%, and two changes of 99.8% ethanol) for 30 min each, as described by Wolny *et al.* (2014). The embryos were embedded in wax (polyethylene glycol distearate and 1-hexadecanol; 9:1 w/w) by passage through a wax:ethanol series (1:2, 1:1, and 2:1 v/v) at 37 °C for 24 h in each solution, followed by one change of pure wax for 24 h. The embryos were transferred to embedding moulds, left to polymerize at RT for 24 h, and sectioned at 5 µm using a Leica RM 2145 microtome. The sections were placed on poly-L-lysine-coated slides and stretched in a drop of water. After drying overnight at RT, the slides were cleared two times for 8 min in 99.8% ethanol and rehydrated in an ethanol in 2×SSC series (90%, 70%, 50%, and 30% v/v), for 8 min each, and finally in 2×SSC for 8 min. The cleared sections were used immediately for fluorescence *in situ* hybridization (FISH).

### Silver staining

The transcriptional activity of 18S–5.8S–25S rDNA sites was determined using a modification of the silver staining method of Hizume *et al.* (1980). Preparations were immersed in 0.01 M borate buffer (0.01 M Na_2_B_4_O_7_; pH 9.2) at RT for 10 min and air-dried. A volume of 50 µl of freshly made 50% aqueous solution of silver nitrate was applied to each slide. To reduce evaporation of the reagent, the slides were covered with nylon meshes and incubated in humid chambers at 42 °C for ~30 min. After several washes in distilled water, the slides were air-dried and mounted in glycerol. After imaging as described below, the slides for FISH were incubated in 4×SSC at 37 °C to remove coverslips and mounting medium, destained by immersing in 30% hydrogen peroxide for 30 s, washed several times in distilled water, and air-dried.

### Immunodetection of 5-methylcytosine

Immunodetection of 5-methylcytosine (5-MeC) was performed according to Borowska *et al.* (2011), using mouse antibodies raised against 5-MeC [Abcam; 1:200 dilution in 1% bovine serum albumin (BSA) in 1×PBS] and goat anti-mouse secondary antibody conjugated with Alexa^488^ (Invitrogen; 1:200 in 1% BSA in 1×PBS). Preparations of meiotic chromosomes were denatured in 70% formamide for 2 min and blocked with 5% BSA before incubation with the primary antibody at 37 °C for 1 h. After three washes for 5 min in 1×PBS, the secondary antibody was applied to the slides, which were then incubated at 37 °C for 1 h. Meiotic chromosomes were counterstained with DAPI in Vectashield. After image acquisition as described below, the slides for FISH were washed in 4×SSC with 0.1% Tween 20 at 37 °C in order to remove coverslips, washed several times in 2×SSC at RT, and subjected to the FISH protocol.

### DNA probes and FISH on squashed meiotic preparations

A 2.3 kb *Cla*I subclone of the 25S rDNA coding region of *A. thaliana* (Unfried and Gruendler, 1990) labelled by nick translation using digoxigenin-11-dUTP (Roche) was used as a probe to detect 35S rDNA gene clusters, according to the method of Idziak *et al.* (2011). The precipitated probe was dissolved in a hybridization mixture consisting of 50% deionized formamide and 20% dextran sulphate in 2×SSC, predenatured at 75 °C for 10 min, and applied to the slide. After denaturation at 75 °C for 4.5 min, meiotic preparations were allowed to hybridize in a humid chamber at 37 °C for 16–20 h. Post-hybridization washes were performed with 10% formamide in 0.1×SSC at 42 °C, which is equivalent to 79% stringency. Immunodetection of digoxigenated probes was performed according to a standard protocol using fluorescein isothiocyanate-conjugated anti-digoxigenin antibodies (Roche), and the chromosomes were counterstained with 2.5 μg/ml DAPI in Vectashield.

### FISH on sections through embryos

The hybridization mixture was prepared and predenatured in the same way as described above for FISH on squashed meiotic preparations. The DNA on the slides was denatured chemically in a denaturation buffer (0.5 M NaOH, 1 M NaCl) at 4 °C for 30 min. After denaturation, slides were washed three times in distilled water at 4 °C for 4 min each and dehydrated in an ethanol series (70%, 90%, and 99.8%). After application of the hybridization mixture to each slide, preparations were allowed to hybridize in a humid chamber at 37 °C for 16–20 h. Post-hybridization washes and DNA counterstaining were performed according to Idziak *et al.* (2011).

### Acrylamide embedding of *B. hybridum* meiocytes

In order to preserve 3D architecture, *B. hybridum* meiocytes were embedded in acrylamide gel, according to the protocol of Bass *et al.* (1997) with some modifications. Fixed anthers at the desired stage of meiosis were collected into 1×Buffer A (2×Buffer A salts, 0.2 mM spermine, 0.5 mM spermidine, 1 mM DTT, and 0.35 M sorbitol) and macerated using a brass rod. A 10 µl suspension of meiocytes was transferred on to a 24 × 24 mm coverslip and mixed with 5 µl of acrylamide/bis-acrylamide combined with 20% ammonium persulfate and 20% sodium persulfate solutions for polymerization. The coverslip with the suspension of meiocytes in acrylamide was covered immediately with another coverslip at a rotational angle of 45° to the first, and the acrylamide solution was allowed to polymerize for 30–60 min. The coverslips were separated with a razor blade and the one with the polyacrylamide pad was transferred to a small Petri dish before FISH.

### FISH on polyacrylamide pads

FISH on the polyacrylamide pads was done as described by Bass *et al.* (1997) with some modifications. The time of each wash was extended to 30 min to ensure an adequate penetration of reagents into the polyacrylamide gel. The predenatured hybridization mix containing the 25S rDNA probe was applied to the pads and denatured together at 75 °C for 8 min. After renaturation for ~20 h, post-hybridization washes were performed in 20% formamide in 0.1×SSC at 37 °C. Pads were mounted in mounting medium [200 mM Tris–HCl, pH 8, 2.3% DABCO (1,4-diazobicyclo(2,2,2)octane), 78% glycerol, and 1 µg/ml DAPI] and stored at 4 °C until imaged.

### Image acquisition and analysis

All images of meiotic chromosomes after silver staining and immunodetection of 5-MeC and sequential FISH experiments were acquired using a Zeiss Axio Imager.Z.2 wide-field fluorescence microscope equipped with an AxioCam HRm monochromatic camera. Meiocytes embedded in polyacrylamide and embryo sections were optically sectioned using an Olympus FV1000 confocal microscope system equipped with a ×60/1.35 PlanApo objective. Image stacks were acquired by traversing from the top to the bottom of a nucleus in 0.2 µm steps. Image processing, including the rendering of the Z-stacks from a series of optical sections of the meiocytes, was performed using ImageJ (https://imagej.nih.gov/ij/; last accessed 10/12/2018).

## Results and discussion

### *B. stacei*-derived 35S rDNA loci do not form a nucleolus in meiocytes and microspores

**Fig. 1.**
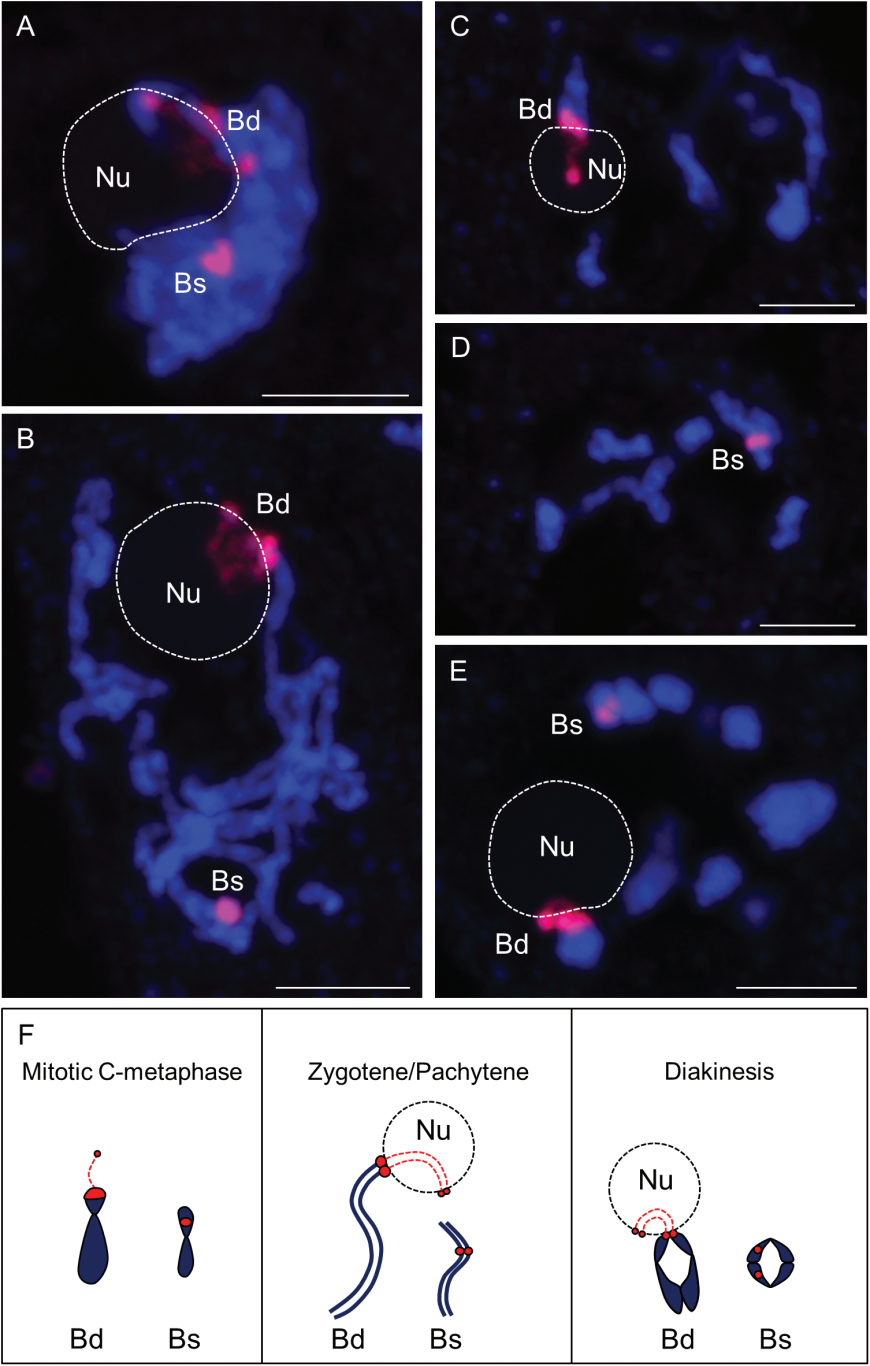
Localization of *B. distachyon*- and *B. stacei*-inherited 35S rDNA loci (red fluorescence) in 3D cytogenetic preparations of *B. hybridum* prophase I meiocytes. Selected sections that contain 25S rDNA hybridization signals are shown. (A) Zygotene. (B) Pachytene. (C, D) Two different sections of one nucleus at diplotene. (E) Diakinesis. (F) Diagram showing the localization and condensation state of 35S rDNA loci in *B. hybridum* at different stages of mitosis and meiosis. Bd, *B. distachyon*-like 35S rDNA loci; Bs, *B. stacei*-like 35S rDNA loci; Nu, nucleolus. Sections are counterstained with DAPI (blue fluorescence). Scale bars=5 µm.

In this study, we addressed the question of whether the ND phenomenon in *B. hybridum* is a developmentally regulated process, having previously shown that ND is present in both meristematic and differentiated cells of *B. hybridum* roots (Idziak and Hasterok, 2008; Borowska-Zuchowska *et al.*, 2016). The localization of 35S rDNA loci inherited from both ancestral species (*B. distachyon* and *B. stacei*) in meiocytes and microspores of *B. hybridum* was revealed by using FISH with a 25S rDNA probe. As the preservation of the 3D architecture of the meiocytes and microspores is crucial in determining the positions of rDNA loci and their association with different structures of the nucleus, such as the nucleolus (or nucleoli), whole cells released from anthers of *B. hybridum* were embedded in a polyacrylamide matrix (Bass *et al.*, 1997; Hurel *et al.*, 2018). A single nucleolus per cell was observed in all substages of prophase I of male meiosis, from zygotene up to late diakinesis (Fig. 1; Supplementary Videos S1–S4 at *JXB* online). Interestingly, only one bivalent with terminally located and partially decondensed 35S rDNA loci was associated with the nucleolus (Fig. 1), while another bivalent with proximally located, highly condensed rDNA loci was not attached to the nucleolus in all prophase I meiocytes (Fig. 1; Supplementary Videos S1–S4). We previously showed that 35S rDNA loci occupy distinct and different positions in the chromosomes of the two ancestral species (Fig. 1F; Hasterok *et al.*, 2004; Borowska-Zuchowska *et al.*, 2016). *B. distachyon*-derived 35S rDNA loci are located at the termini of the short arms of chromosome pair Bd5, whereas the loci inherited from the other ancestor are present in the proximal regions of the other, significantly smaller, chromosome pair Bs10 (Borowska-Zuchowska *et al.*, 2016; Lusinska *et al.*, 2018). On the basis of these data, we can conclude that only the 35S rDNA loci inherited from the *B. distachyon*-like ancestor are associated with the nucleolus during meiotic prophase I, providing indirect evidence of their transcriptional activity (Fig 1A–E; Bd bivalents).

**Fig. 2.**
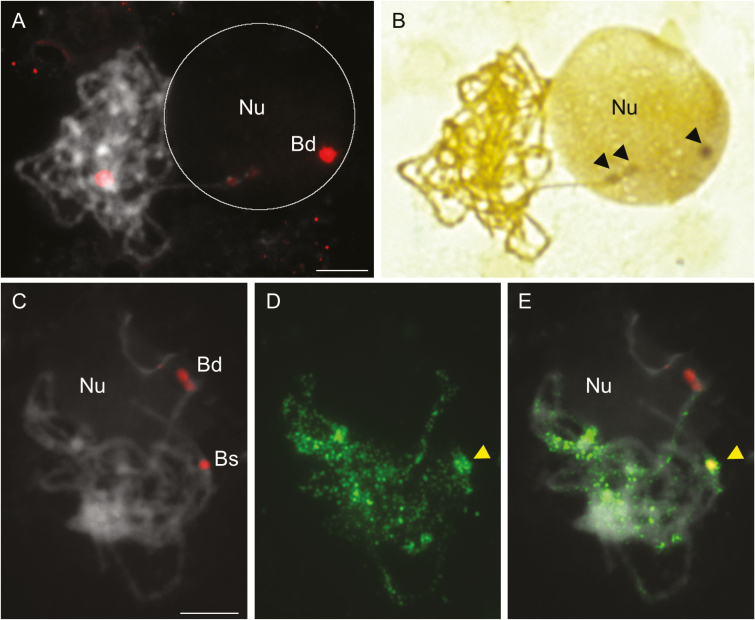
Transcriptional activity and DNA methylation pattern of 35S rDNA loci in *B. hybridum* meiocytes at pachytene. (A, B) Sequential FISH with 25S rDNA (red fluorescence) as a probe (A) and silver staining (B) on a representative cell at pachytene. Black arrows indicate Ag-NORs. (C–E) Sequential FISH with 25S rDNA (red fluorescence) as a probe (C) and immunolocalization of 5-MeC (D) on a representative pachytene. (E) Superimposed images C and D. Yellow arrows indicate the position of *B. stacei*-inherited 35S rDNA loci. Bd, *B. distachyon*-like 35S rDNA loci; Bs, *B. stacei*-like 35S rDNA loci; Nu, nucleolus. Bivalents are counterstained with DAPI (grey). Scale bars=5 µm.

To assess the transcriptional activity of the rDNA loci inherited from the other ancestor in a more direct way, sequential silver staining followed by FISH with a 25S rDNA probe were applied to meiotic spreads of *B. hybridum*. Ag-NOR bands colocalized with the *B. distachyon*-like 35S rDNA loci only (Fig. 2A, B), implying that ND in *B. hybridum* is active during prophase I of meiosis. To determine whether DNA methylation is responsible for the selective suppression of *B. stacei*-inherited rDNA loci, we immunodetected 5-MeC and performed FISH with a 25S rDNA probe on pachytene spreads of *B. hybridum*. Fig. 2C–E shows that the rDNA loci from both progenitors show different levels of DNA methylation; the *B. distachyon*-originated loci have relatively weak anti-5-MeC signals (Fig. 2C–E; bivalent Bd), and the *B. stacei*-like loci have a very high density of 5-MeC foci (Fig. 2C–E; bivalent Bs). Similar differences in the DNA methylation patterns of rDNA loci were observed in mitotic metaphase chromosomes and interphase nuclei from root tip cells of *B. hybridum* (Borowska-Zuchowska and Hasterok, 2017). The relatively high level of DNA methylation of *B. stacei*-inherited rDNA loci in both somatic (root tip cells) and generative (meiocytes) cells of *B. hybridum* indicates the involvement of this epigenetic modification in the establishment and maintenance of ND in this species. It was demonstrated that the level of DNA methylation, especially in the PolI promoter sites, directly correlates with the expression of 35S rRNA genes. The actively transcribed rDNA units are hypomethylated, as has been reported for *Brassica* (Ksiazczyk *et al*., 2011), wheat (Carvalho *et al.*, 2010), *Solanum* (Komarova *et al.*, 2004) and *Tragopogon* (Dobesova *et al*., 2015; Matyasek *et al.*, 2016) species. For instance, in the allotetraploid *Tragopogon mirus* (genomes DDPP), which has a homozygous macrodeletion of the majority of the D genome-originated 35S rDNA repeats, it was observed that the underdominant P genome-like rDNA units were reactivated. Their transcriptional activation was accompanied by a reduced level of DNA methylation, especially at the symmetrical CG and CHG nucleotide motifs in the PolI promoters. In spite of the codominance of the P and D genome-like 35S rDNA in the roots, flowers, and calli, the dominance of the rRNA genes from the D genome was observed in the leaf tissue (Dobesova *et al*., 2015). Such an expression pattern of rRNA genes was also observed in artificial *Solanum* allopolyploids in which reactivation of the underdominant rRNA genes was found in the anthers, thus indicating the developmental modulation of ND (Komarova *et al.*, 2004).

Studies of dicot and monocot interspecific and intergeneric hybrids and allopolyploids exhibiting ND have emphasized the developmental regulation of this phenomenon (Castilho *et al.*, 1995; Silva *et al.*, 1995; Chen and Pikaard, 1997*b*; Pontes *et al.*, 2007). Chen and Pikaard (1997*b*) revealed that rRNA transcripts from only one progenitor were detected in vegetative tissues in both natural and resynthesized *Brassica napus,* with the exception of root meristematic cells, which have codominant *Brassica rapa*- and *Brassica oleracea*-like rRNA genes (Hasterok and Maluszynska, 2000). However, the less-expressed *B. oleracea*-like genes are transcriptionally reactivated after the transition from the vegetative to the generative phase, in which rRNA transcripts from both progenitors are present in all floral organs, including sepals, petals, anthers, and siliques (Chen and Pikaard, 1997*b*). It has been shown in many plant and animal species that the transcription of 35/45S rRNA genes is accelerated during prophase I of meiosis (Schultz and Leblond, 1990; Stahl *et al.*, 1991; Smolinski *et al*., 2007; Kolowerzo-Lubnau *et al*., 2015). If ND is one of the mechanisms of rRNA gene dosage compensation in allopolyploids, the lack of this phenomenon at particular stages of development, such as in root tip cells and floral organs, may reflect the greater need of these cells to produce ribosomes in order to support an intensive demand for proteins. However, more recent studies of different cultivars of *B. napus* revealed that developmental regulation of ND may even be genotype specific, as in most *B. napus* accessions only trace expression of *B. oleracea*-derived rRNA genes was detected in flower buds (Sochorova *et al*., 2017).

**Fig. 3.**
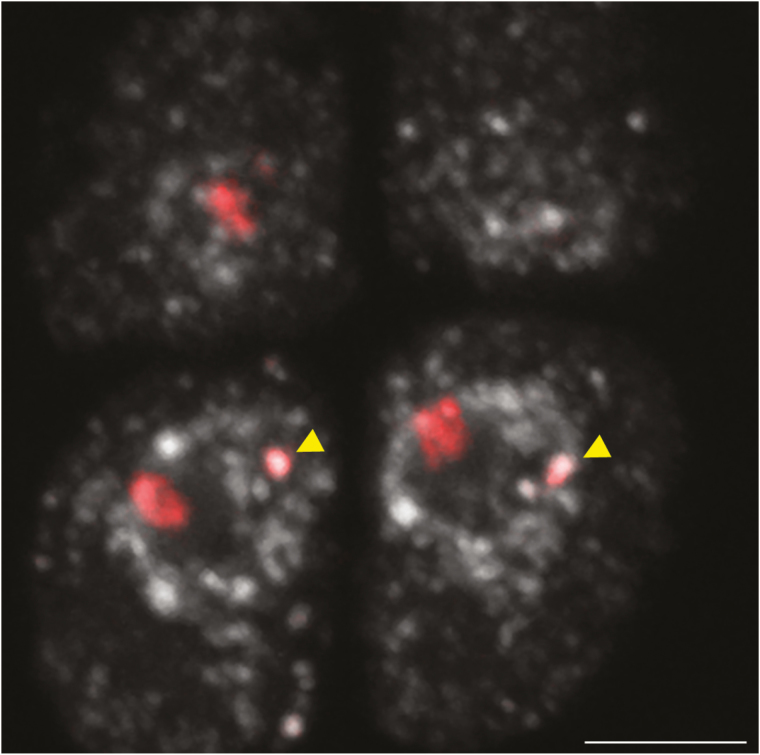
Localization of *B. distachyon*- and *B. stacei*-inherited 35S rDNA loci (red fluorescence) in 3D cytogenetic preparations of *B. hybridum* microspores. Selected sections containing 25S rDNA hybridization signals are shown. Yellow arrows indicate the position of *B. stacei*-inherited 35S rDNA loci. Sections are counterstained with DAPI (grey). Scale bars=5 µm.

Because ND is a fully reversible process that has an epigenetic basis, the transcriptional activation of the underdominant rRNA gene set may be related to changes in epigenetic status, associated with, for example, DNA hypomethylation and demethylation/hyperacetylation of histones. It was shown that *B. oleracea*-derived rDNA loci can be reactivated even in vegetative tissues of *B. napus* following either global DNA hypomethylation by 5-AzadC treatment or histone hyperacetylation after TSA treatment (Chen and Pikaard, 1997*a*). ND is also abolished after 5-AzadC and/or TSA treatment in vegetative tissues of other allopolyploids and hybrids, such as *A. suecica* (Chen *et al.*, 1998), triticale (Amado *et al.*, 1997), and wheat × rye intergeneric hybrids (Neves *et al.*, 1995; Vieira *et al.*, 1990). The DNA hypomethylation of the *B. hybridum* genome by 5-AzaC treatment, however, seems to be insufficient for activation of the underdominant *B. stacei*-like rDNA loci. Taking together our present results, the fact that *B. stacei*-like loci are not reactivated by hypomethylation (Borowska-Zuchowska and Hasterok, 2017), our previous analysis of the 35S rDNA intergenic spacers in *B. hybridum* (Borowska-Zuchowska *et al.*, 2016), and the presence of at least one *B. hybridum* genotype (ABR117) in which *B. stacei*-inherited rDNA loci are eliminated (Hasterok *et al.*, 2004; Borowska-Zuchowska *et al.*, 2016), we speculate that underdominant 35S rRNA genes may have evolved as pseudogenes and be progressively eliminated during the evolution of this allotetraploid. If such a scenario holds true for *B. hybridum,* the *B. stacei*-inherited 35S rRNA gene set may be silenced permanently owing to changes at the DNA level. Such a pseudogenization of 35S rDNA was found in the gymnosperm *Cycas revoluta*, in which only a minority (~20%) of the rRNA genes remained functional. The rest of the rDNA repeats accumulated mutations, mainly in the CG and CHG sequence contexts, which affected both the non-coding and coding regions (Wang *et al.*, 2016). In most of the species studied to date, however, the units that are present in the 35S rDNA array have been homogenous owing to their concerted evolution via an unequal crossing-over and/or gene conversion. In allopolyploid species, the replacement of ancestral rDNA variants may be caused by an intergenomic homogenization (Kovarik *et al.*, 2004, 2008; Mahelka *et al.*, 2013; Volkov *et al.*, 2017). In our previous work, the *B. stacei*-specific IGS variant that was used as the probe in FISH gave signals only in the *B. stacei*-originated chromosomes; thus, a complete replacement of the *B. stacei*-like rDNA units by the *B. distachyon*-like ones in *B. hybridum* can be ruled out (Borowska-Zuchowska *et al.*, 2016). In some allopolyploids, such as *Nicotiana* species from the sections *Polydicliae* and *Repandae*, both a reduction in the rDNA loci number to diploid-like numbers (Clarkson *et al.*, 2005) and new rDNA families (Lim *et al.*, 2007) were found. In *B. hybridum,* genotypes that had either a significant reduction in the *B. stacei*-like rDNA copy number (ABR100 and ABR107) or the elimination (ABR117) of the aforementioned loci were observed, despite the fact that this species is considered to be a relatively young allopolyploid that arose no more than ~1 million years ago (Catalan *et al*., 2012). Thus, the molecular structure and evolution of the transcriptionally silenced *B. stacei*-like rDNA loci in *B. hybridum* still need to be verified at the sequence level using novel next-generation sequencing strategies. Preferential silencing of rDNA loci via ND may be erased after meiotic reprogramming. For example, the 35S rRNA gene set of rye origin in hexaploid triticale is suppressed in root meristematic cells and during the first meiotic division, but is then transcriptionally activated in microspores (Silva *et al.*, 1995). In this study, we verified the distribution of 35S rDNA loci in the nuclei of *B. hybridum* microspores. As expected, we observed two hybridization signals corresponding to 35S rDNA in haploid microspores after FISH, which differed significantly in condensation. One signal is characterized by a dispersed chromatin structure and probably corresponds to the *B. distachyon*-like rDNA locus, and the other is within a chromocentre at the nuclear periphery and is highly condensed (Fig. 3; Supplementary Video S5). This distribution of 25S rDNA FISH signals is similar to that in somatic nuclei of *B. hybridum* roots (Borowska-Zuchowska *et al.*, 2016; Borowska-Zuchowska and Hasterok, 2017), suggesting that ND is not abolished in *B. hybridum* cells even after meiotic division.

### ND is established in all embryonic tissues of *B. hybridum*

**Fig. 4.**
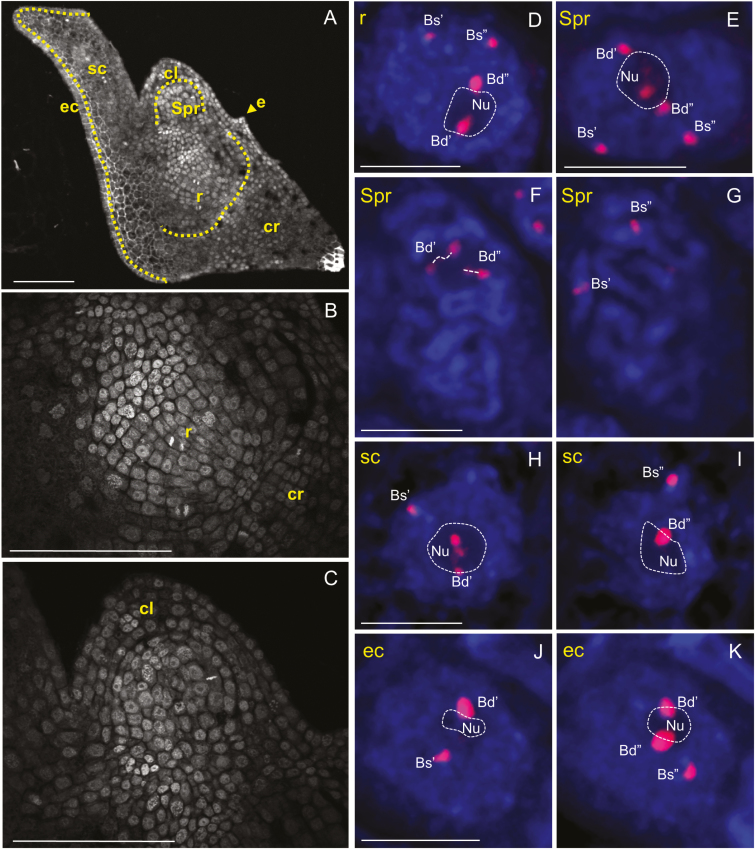
Distribution of 35S rDNA loci in nuclei/prometaphase chromosomes from different tissues of the *B. hybridum* embryo at BBCH83. (A) Longitudinal cross-section through a whole embryo. (B) Enlargement of the radicle and a fragment of coleorhiza. (C) Enlargement of shoot primordium and coleoptile. (D–K) FISH with 25S rDNA (red fluorescence) as a probe in nuclei from different embryo tissues. Selected sections that contain 25S rDNA hybridization signals and one or more nucleoli are presented. Bd, *B. distachyon*-like 35S rDNA loci; Bs, *B. stacei*-like 35S rDNA loci; cl, coleoptile; cr, coleorhiza; e, epiblast; ec, epithelial cells; Nu, nucleolus; r, radicle; sc, scutellum; Spr, shoot primordium. Sections are counterstained with DAPI (blue fluorescence). Scale bars (A–C)=50µm; (D–K)=5 µm.

**Fig. 5.**
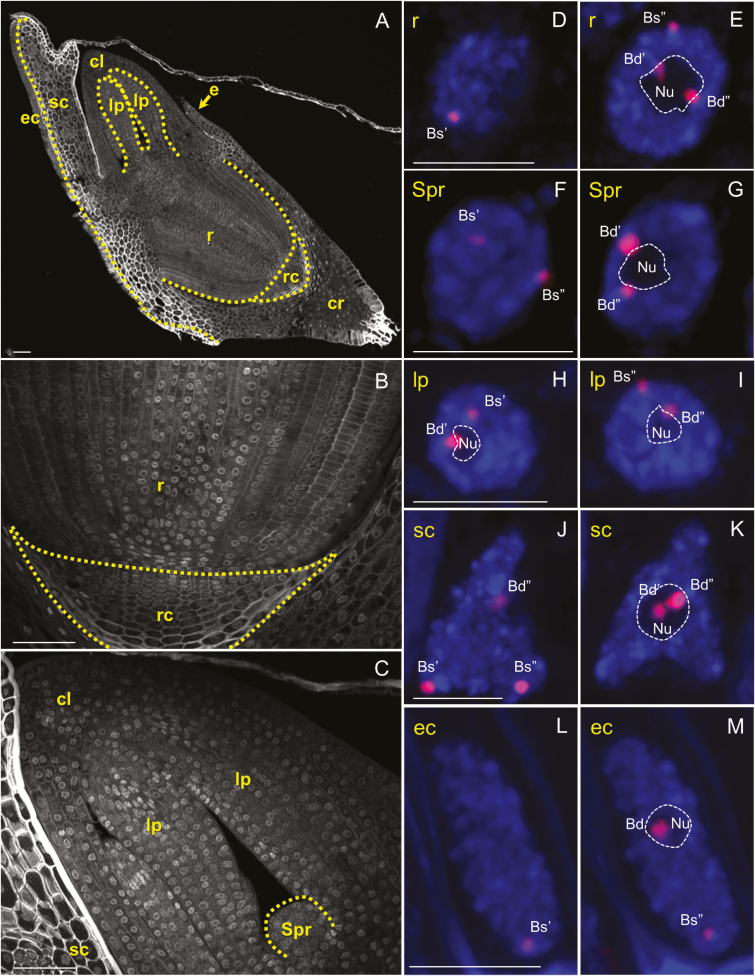
Distribution of 35S rDNA loci in nuclei from different tissues of the *B. hybridum* embryo at BBCH01. (A) Longitudinal cross-section through a whole embryo. (B) Enlargement of the radicle and root cap. (C) Enlargement of the shoot primordium, leaf primordia, and coleoptile. (D–M) FISH with 25S rDNA (red fluorescence) as a probe in nuclei from different embryo tissues. Selected sections that contain 25S rDNA hybridization signals and one or more nucleoli are presented. Bd, *B. distachyon*-like 35S rDNA loci; Bs, *B. stacei*-like 35S rDNA loci; cl, coleoptile; cr, coleorhiza; e, epiblast; ec, epithelial cells; lp, leaf primordia; Nu, nucleolus; r, radicle; rc, root cap; sc, scutellum; Spr, shoot primordium. Sections are counterstained with DAPI (blue fluorescence). Scale bars (A–C)=50µm; (D–M)=5 µm.

We determined the positions of 35S rDNA loci in nuclei from *B. hybridum* embryos at the BBCH83 (Fig. 4; Supplementary Videos S6, S7, S9, S10) and BBCH01 (Fig. 5; Supplementary Videos S11–S15) stages. Surprisingly *B. stacei*-originated loci are located close to the nuclear envelope (Figs 4 and 5) in the nuclei of all embryonic tissues (radicle, shoot primordium, coleoptile, scutellum with epithelial cells, and leaf primordia), usually in DAPI-positive regions. We showed that only *B. distachyon*-like loci are able to form the nucleolus/nucleoli in cells derived from both BBCH83 and BBCH01 embryos (Figs 4 and 5; Supplementary Videos S6, S7, S9, S10–S15), as was also shown in somatic nuclei isolated from *B. hybridum* roots (Borowska-Zuchowska *et al.*, 2016). In mitotic chromosome spreads of embryo cells, secondary constrictions were consistently formed by *B. distachyon*-derived chromosomes only (Fig. 4F, G; Supplementary Video S8).

The timing of the establishment of ND during the life cycle can vary significantly between different allopolyploid organisms (Castilho *et al.*, 1995; Pontes *et al.*, 2007). For example, ND is re-established in every generation during early postembryonic development in *A. suecica* (Pontes *et al.*, 2007). Both molecular and cytogenetic studies have revealed that *A. thaliana*-originated 35S rRNA genes are progressively silenced in tissues derived from shoot and root apical meristems of *A. suecica* seedlings by progressive condensation and heterochromatization of these loci. Interestingly, *A. thaliana*-like rRNA genes are still transcriptionally active and therefore associated with the nucleolus even 4 days after germination (Pontes *et al.*, 2007). However, the timing of ND establishment in monocotyledonous allopolyploids seems to be different from that in the dicot *A. suecica.* The presence of fully established ND in root meristematic cells 2–3 days after germination was detected in allopolyploid triticale (Lacadena *et al.*, 1984; Silva *et al.*, 1995) and in root tip cells derived from 3-day-old *B. hybridum* seedlings (Idziak and Hasterok, 2008).

The inactive state of *B. stacei*-inherited rDNA loci at different stages of embryo development supports our hypothesis of the gradual accumulation of mutations in inactive *B. stacei*-like rDNA loci. However, the establishment of ND during earlier stages of *B. hybridum* embryo development cannot be excluded, as there is evidence that the pattern of rRNA gene expression in grasses may be reprogrammed during earlier stages of embryo development. For example, the rRNA genes of rye origin in wheat × rye F1 hybrids are transcriptionally silenced between days 4 and 5 after fertilization (Castilho *et al.*, 1995), which is much earlier than in the dicot *A. suecica*. Thus, investigation of rDNA activity during the earlier stages of *B. hybridum* embryo formation is still worth pursuing. Moreover, the complex analysis of rRNA gene loci in resynthesized *B. hybridum*, which has been obtained recently (Dinh Thi *et al.*, 2016), may shed some new light on the enforcement and maintenance of ND in grass allopolyploids.

## Supplementary data

Supplementary data are available at *JXB* online.

Video S1. 3D distribution of 25S rDNA hybridization signals (red fluorescence) in the *B. hybridum* meiocyte at zygotene from Fig. 1A.

Video S2. 3D distribution of 25S rDNA hybridization signals in the *B. hybridum* meiocyte at pachytene from Fig. 1B.

Video S3. 3D distribution of 25S rDNA hybridization signals in the *B. hybridum* meiocyte at diplotene from Fig. 1C, D.

Video S4. 3D distribution of 25S rDNA hybridization signals in the *B. hybridum* meiocyte at diakinesis from Fig. 1E.

Video S5. 3D distribution of 25S rDNA hybridization signals in the *B. hybridum* tetrad of microspores from Fig. 3.

Video S6. 3D distribution of 25S rDNA hybridization signals in a representative nucleus from the radicle of a *B. hybridum* embryo at BBCH83 from Fig. 4D.

Video S7. 3D distribution of 25S rDNA hybridization signals in representative nuclei from the shoot primordium of a *B. hybridum* embryo at BBCH83; the nucleus shown in Fig. 4E is delimited by a white rectangle.

Video S8. 3D distribution of 25S rDNA hybridization signals in a representative prometaphase from the shoot primordium of a *B. hybridum* embryo at BBCH83 from Fig. 4F, G.

Video S9. 3D distribution of 25S rDNA hybridization signals in a representative nucleus from the scutellum of a *B. hybridum* embryo at BBCH83 from Fig. 4H, I.

Video S10. 3D distribution of 25S rDNA hybridization signals in a representative nucleus from an epithelial cell of a *B. hybridum* embryo at BBCH83 from Fig. 4J, K.

Video S11. 3D distribution of 25S rDNA hybridization signals in representative nuclei from the radicle of a *B. hybridum* embryo at BBCH01; the nucleus at the bottom is shown in Fig. 5D, E.

Video S12. 3D distribution of 25S rDNA hybridization signals in representative nuclei from the shoot primordium of a *B. hybridum* embryo at BBCH01; the nucleus in Fig. 5F, G is identified by a white rectangle.

Video S13. 3D distribution of 25S rDNA hybridization signals in representative nuclei from the leaf primordia of a *B. hybridum* embryo at BBCH01; the nucleus in Fig. 5H, I is identified by a white rectangle.

Video S14. 3D distribution of 25S rDNA hybridization signals in a representative nucleus from the scutellum of a *B. hybridum* embryo at BBCH01 from Fig. 5J, K.

Video S15. 3D distribution of 25S rDNA hybridization signals in representative nuclei from the epithelial cells of a *B. hybridum* embryo at BBCH01; the nucleus from Fig. 5L, M is identified by a white rectangle.

Supplementary Video S1

Supplementary Video S2

Supplementary Video S3

Supplementary Video S4

Supplementary Video S5

Supplementary Video S6

Supplementary Video S7

Supplementary Video S8

Supplementary Video S9

Supplementary Video S10

Supplementary Video S11

Supplementary Video S12

Supplementary Video S13

Supplementary Video S14

Supplementary Video S15

Supplementary_Video_Legends

## Author contributions

NBZ, ER, and RH conceived and designed the study; NBZ, ER, EW, and AB performed the experiments; NBZ, ER, and RH analysed the data; NBZ, ER, and RH wrote the paper.
