# Supplementary material for: Ribosomal DNA loci derived from Brachypodium stacei are switched off for major parts of the life cycle of Brachypodium hybridum
Source: J Exp Bot. 2018 Nov 27;70(3):805–15. doi: 10.1093/jxb/ery425 (PMC6363085; doi:10.1093/jxb/ery425)
Supplement: Supplementary_Video_Legends [file ery425_suppl_supplementary_video_legends.docx]

**SUPPLEMENTARY DATA**

**Video S1.**

Three-dimensional distribution of 25S rDNA hybridisation signals (red fluorescence) in the *B. hybridum* meiocyte at zygotene from Fig. 1A.

**Video S2.**

Three-dimensional distribution of 25S rDNA hybridisation signals (red fluorescence) in the *B. hybridum* meiocyte at pachytene from Fig. 1B.

**Video S3.**

Three-dimensional distribution of 25S rDNA hybridisation signals (red fluorescence) in the *B. hybridum* meiocyte at diplotene from Fig. 1C1-C2.

**Video S4.**

Three-dimensional distribution of 25S rDNA hybridisation signals (red fluorescence) in the *B. hybridum* meiocyte at diakinesis from Fig. 1D.

**Video S5.**

Three-dimensional distribution of 25S rDNA hybridisation signals (red fluorescence) in the *B. hybridum* tetrad of microspores from Fig. 3.

**Video S6.**

Three-dimensional distribution of 25S rDNA hybridisation signals (red fluorescence) in a representative nucleus from the radicle of a *B. hybridum* embryo at BBCH83 from Fig. 4D.

**Video S7.**

Three-dimensional distribution of 25S rDNA hybridisation signals (red fluorescence) in representative nuclei from the shoot primordium of a *B. hybridum* embryo at BBCH83. The nucleus shown in Fig. 4E is delimited by a white rectangle.

**Video S8.**

Three-dimensional distribution of 25S rDNA hybridisation signals (red fluorescence) in a representative prometaphase from the shoot primordium of a *B. hybridum* embryo at BBCH83 from Fig. 4F1-F2.

**Video S9.**

Three-dimensional distribution of 25S rDNA hybridisation signals (red fluorescence) in a representative nucleus from the scutellum of a *B. hybridum* embryo at BBCH83 from Fig. 4G1-G2.

**Video S10.**

Three-dimensional distribution of 25S rDNA hybridisation signals (red fluorescence) in a representative nucleus from an epithelial cell of a *B. hybridum* embryo at BBCH83 from Fig. 4H1-H2.

**Video S11.**

Three-dimensional distribution of 25S rDNA hybridisation signals (red fluorescence) in representative nuclei from the radicle of a *B. hybridum* embryo at BBCH01. The nucleus at the bottom is shown in Fig. 5D1-D2.

**Video S12.**

Three-dimensional distribution of 25S rDNA hybridisation signals (red fluorescence) in representative nuclei from the shoot primordium of a *B. hybridum* embryo at BBCH01. The nucleus in Fig. 5E1-E2 is identified by a white rectangle.

**Video S13.**

Three-dimensional distribution of 25S rDNA hybridisation signals (red fluorescence) in representative nuclei from the leaf primordia of a *B. hybridum* embryo at BBCH01. The nucleus in Fig. 5F1-F2 is identified by a white rectangle.

**Video S14.**

Three-dimensional distribution of 25S rDNA hybridisation signals (red fluorescence) in a representative nucleus from the scutellum of a *B. hybridum* embryo at BBCH01 from Fig. 5G1-G2.

**Video S15.**

Three-dimensional distribution of 25S rDNA hybridisation signals (red fluorescence) in representative nuclei from the epithelial cells of a *B. hybridum* embryo at BBCH01. The nucleus from Fig. 5H1-H2 is identified by a white rectangle.
